# Clinical Effect of Acupuncture on Endemic Skeletal Fluorosis: A Randomized Controlled Trial

**DOI:** 10.1155/2013/839132

**Published:** 2013-11-17

**Authors:** Zhou Jincao, Wu Zhongchao, Chen Zhongjie, Zhao Xiaoguang, Hu Jing, Jiao Yue, Li Guiran, Pang Li

**Affiliations:** Acupuncture and Moxibustion Institute of China Academy of Chinese Medical Science, Beijing 100700, China

## Abstract

*Objective*. To evaluate the effect of acupuncture on endemic skeletal fluorosis (ESF) through the randomized controlled trial. *Methods*. Ninety-nine cases were divided into the treatment group (68 cases) and the control group (31 cases) randomly. Normal acupuncture combined with electroacupuncture was used in treatment group, while Caltrate with vitamin D tablets were applied in control group. After 2 courses, the VAS, urinary fluoride, serum calcium, and serum phosphate were evaluated before and after treatment. *Results*. Both of these two methods could relieve pain effectively and the effect of acupuncture was better (*P* < 0.05). In treatment group, the content of urinary fluoride after treatment was higher than before (*P* < 0.05), while the content of serum calcium and phosphate was lower (*P* < 0.05). *Conclusion*. The effect of acupuncture on relieving pain and promoting discharge of urinary fluoride is better than that of western medicine. Acupuncture can reduce the content of serum calcium and phosphate.

## 1. Introduction

Endemic fluorosis, known as endemic skeletal fluorosis (ESF), is a kind of chronic poisoning disease caused by excessive intake of fluorine for a long time during people's lives. It usually includes dental and skeletal fluorosis. The disease, endemic skeletal fluorosis in drink-water type, is caused by high content of fluorine in drinking water in prevalent areas. At present, the pandemic disease has affected 35 countries and regions.

The pathogenesis of skeletal fluorosis is unclear, so the method of descending fluorine though water improvement is the only effective one [1, 2], but it needs a long time for the elimination of fluorine in body and the improvement of clinical symptoms. Drug therapy usually includes chemical elements such as calcium, magnesium, and aluminum, vitamins, and amino acid [3]. Though these drugs can produce certain effect, their unstable effect and side effects still should be considered.

This study applies acupuncture therapy to treat skeletal fluorosis, which promotes qi circulation, regulates meridians, dredges collaterals to relieve pain, and improves the joint motion. The mechanism of acupuncture analgesia has been studied profoundly and gets a positive effect. The dysfunction of joints actually belongs to a fibrous ankylosis, a reversible change, which results from low tensity of muscle, tendon, and ligament, around the affected joint after pain relief [4]. The calcium can coordinate with fluorion in the intestinal tract, and the coordination compound-calcium fluoride is excreted to lower the absorption of fluorion; on the other hand, the diet structure with profuse calcium is suitable for patients with skeletal fluorosis [5].

The study is designed as a randomized controlled trial and obeys the scientific research principle of random, blindness, control, and so forth, also follows the TCM treating principle of promoting blood circulation to remove blood stasis, dispelling wind to eliminate dampness, and dredging collaterals to resolve pain, and aims to evaluate the acupuncture treatment on skeletal fluorosis for 99 patients by means of clinical outcomes as pain alleviation, urinary fluoride, serum phosphate, and serum calcium.

## 2. Materials and Methods

### 2.1. General Data

From May 2009 to June 2010, the study enrolled 99 patients suffering from skeletal fluorosis diagnosed by WS 192–2008 health industry standard of China. All of them lived in Caosi village, Qing country, Cangzhou, Hebei province (the fluorine content in water from 2.67 to 7 mg/L).

### 2.2. Inclusive and Exclusive Criteria

Inclusive criteria are as follows:

1. age from 30 to ~75 years old (including 30 and 75 years old);
2. the syndrome which is blood stasis and phlegm and dampness blockage (the detailed syndrome differentiation can be referred in the addendum);
3. life history with long-time consuming water of high-content fluorine;
4. in accordance with WS 192-2008 health industry standard of China;
5. patients who signed the informed consent volunteer.

Exclusive criteria are as follows:

1. the etiology that was due to burning the coal with high fluoride or drink the brick-tea water with high fluoride content;
2. the disease that is at the severe stage, and also patients suffers from bedridden status, paralysis, or cannot live without others;
3. complications of severe diseases from primary cardiocerebral vascular, hepatic, nephritic, hemopoietic, and nervous systems, drug addicts, or psychopaths;
4. patients suffering from hepatitis B, tuberculosis, and malignancy;
5. women with pregnancy or lactation;
6. trauma of bone or joints within 2 months, without fully recovery;
7. continuous intake of medicine for tonifying kidney or calcium supplement 3 months before treatment;
8. intake of purified or cleaned water with normal content of fluoride;
9. severe adverse reaction to acupuncture, Chinese herbal medicine, and western medicine of the study (eg., fainting during acupuncture, drug allergy).

### 2.3. Method

The randomized controlled method was conducted, and the stratification method was also employed with severity of disease as the layering factor. The ratio of patients assigned to the treatment and control group was 2 : 1, and the sequence number was generated and kept in the randomized center. Finally, the treatment group consisted of 27 male patients and 42 female patients (3 patients dropped off), with age 54.76 ± 10.22 years old; the control group consisted of 12 male patients and 19 female patients (5 patients dropped off), with average age of 60.03 ± 9.44 years old.

The treatment group (acupuncture group) based on the TCM treating principle of promoting blood circulation to remove blood stasis, dispelling wind and eliminating dampness, as well as dredging collaterals to resolve pain, employed the acupuncture treatment with electroacupuncture. The basic points group was combined with local points group.

*The Basic Points.* The basic points are Dazhui (Du 17), Geshu (BL 17), Quchi (LI 11), Hegu (LI 4), Xuehai (SP 12), Yinlingquan (SP 10), Sanyinjiao (SP 6), and Fenglong (ST 40).

*Local Points*. Local point is added in the case of pain in the local part (shoulder: Jianyu (LI 15); elbow: Chize (LU 5), wrist: Yangchi (SJ 4); neck: Tianzhu (BL 10); waist: Yaoyangguan (DU 3); hip: Huantiao (GB 30) or Juliao (GB 29); knee: Dubi (ST 35); ankle: Jiexi (ST 41)). And a local Ashi point was also combined.

*Points Applied with Electroacupuncture.* The basic points are Quchi (LI 11) and Hegu (LI 4) in the same side, Quchi (LI 11) and Hegu (LI 4) in the same side, and local points in both sides. The dilatational wave was applied.

*The Frequency of Treatment*. Once every other day, 30 min for each time, and points without electroacupuncture were applied with lifting and thrusting and rotating manipulation by hand for 1 min every 15 min, with reducing method. The treatment was applied 3 times a week, 1 course lasted for 1 month, and 2 courses in total.

The control group (Caltrate with vitamin D tablets group) based on rectifying the disorder of calcium-phosphorus metabolism and enhancing the fluoride dispelling applied Caltrate with Vitamin D tablets (Tianjin Wyeth Pharmaceutical, 600 mg/tablet, 60 tablets/bottle) to patients. The oral intake method was 600 mg one time, twice a day, one in morning, and the other in the evening. One course lasted for 1 month and 2 courses in total. The study was approved by the Ethics Committee of the Institute of Acupuncture and Moxibustion, China Academy of Chinese Medical Sciences, and written informed consent was obtained from all the patients.

### 2.4. Clinical Outcomes

#### 2.4.1. VAS Scores of Joint Pain

**Figure 1:**
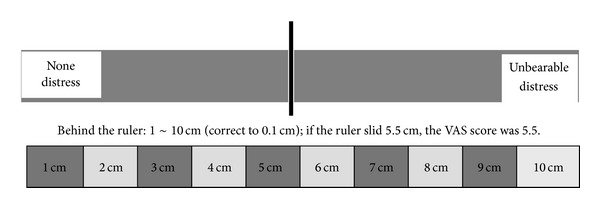
The VAS ruler. VAS scores were measured before and after treatment in both groups.

Visual Analogue Scale (VAS) measured the pain intensity with a 10 cm scale ruler. One end was marked with no pain, and the other end with worst pain. A moving ruler slid from 0 to ~10 in the front, and 0~10 scale numbers just corresponded in the back. Patients were asked to slide the ruler to the position that symbolized the pain intensity for themselves, and the observer got the corresponding scale number in the behind to be the pain score. In front of the ruler, there was no mark, the left end was no pain, and the right end was the worst pain (Figure 1).

#### 2.4.2. Urinary Fluoride

Urinary fluoride was measured by electrode method using the Urinary Fluoride Tester (HACH-F523) in the laboratory department of Caosi Center Hospital of Hebei Province. The analysis and calculation was based on the standard curve method (WS/T 89-1996 health industry standard of China). Urinary fluoride was measured before and after treatment in both groups.

#### 2.4.3. Serum Phosphorus and Serum Calcium

*Serum Calcium.* Methyl thyme phenol blue colorimetric method (MTB): the adult: 2.03~2.54 mmol/L; children: 2.25~2.67 mmol/L.

*Serum Phosphorus.* Molybdenum acid salt method: the adult: 0.97~1.45 mmol/L, children: 1.45~2.1 mmol/L.

Both of them were measured in the laboratory department of Caosi Center Hospital of Hebei Province.

#### 2.4.4. Statistic Analysis

SPSS 17.0 was adopted to analyze data. According to the clinical data and certain statistical principle, the baseline, therapeutic effect, and safety were analyzed and expressed by mean ± standard deviation for measurement data. Measurement data was dealt by normality test, and if it was in accordance with normal distribution, *t* test would be employed then; if the measurement data was not in accordance with normal distribution, parametric test would be employed after data altered to normal change. If the data was not in accordance with normal distribution, rank sum test was adopted. The rank sum test was also adopted for the ranked data. *P* < 0.05 (bilateral sides) meant comparison between groups had statistical significance.

## 3. Results and Discussion

### 3.1. Results

#### 3.1.1. General Data

There was no statistical significance between the water fluoride in residential area, time of residence, and labor intensity of two groups (*P* > 0.05) (Tables 1(a) to 1(c)). 

#### 3.1.2. VAS

**Figure 2:**
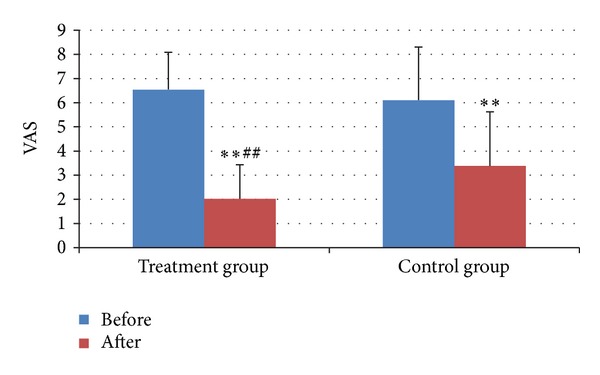
VAS before and after treatment in two groups. **P < 0.01, compared with that before treatment; ^##^ *P* < 0.01, compared with control group.

The VAS after treatment in two groups both decreased than those before treatment (both *P* < 0.01) (Figure 1). The effect on decreasing VAS in treatment group was better than that in control group, and the difference was statistically significant (*P* < 0.01) (Figure 2).

#### 3.1.3. Urinary Fluoride

**Figure 3:**
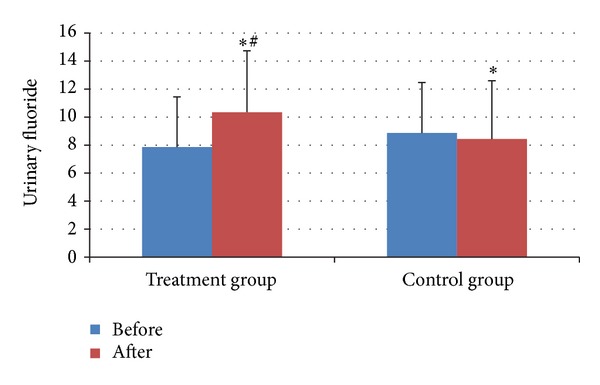
Urinary fluoride before and after treatment in two groups. *P < 0.05, compared with that before treatment; ^#^ *P* < 0.05, compared with control group.

The urinary fluoride after treatment was higher than that before in treatment group (Figure 3). The urinary fluoride after treatment in treatment group was higher than that in control group. And both of the differences were statistically significant (both *P* < 0.05) (Figure 3). 

#### 3.1.4. Serum Calcium and Phosphorus

**Figure 4:**
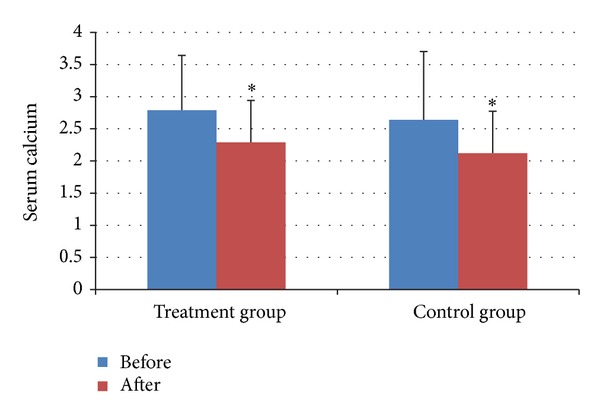
Serum Calcium before and after treatment in two groups. *P < 0.05, compared with that before treatment.

**Figure 5:**
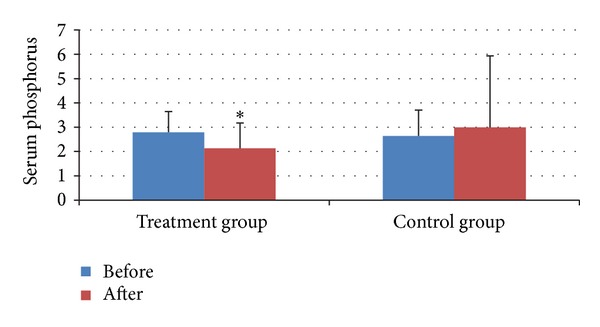
Serum Phosphorus before and after treatment in two groups. *P < 0.05, compared with that before treatment.

The serum calcium after treatment was lower than that before in both groups (Figure 4). The serum phosphorus after treatment was lower than that before in treatment group (Figure 5). And all of the differences were statistically significant (all *P* < 0.05).

### 3.2. Discussion

Though there are lots of western therapy and medicine for treating skeletal fluorosis, the effect of chemical elements preparation (calcium, magnesium, aluminum, etc.), vitamin, and amino acid [3], antioxidant, and anti-inflammatory analgesic dugs is limited and their effect on reliving pain, improving motion of joint, and discharging of urinary fluoride is not so good caused by unclear pathogenesis. Anti-inflammatory analgesic drugs are not suitable to use for a long time. Operation only can be used in its indications, and most patients in remote mountainous areas cannot pay for it. Improvement of water decreasing fluorine is a responsible approach to treat drinking water type of skeletal fluorosis, while the necessary government and economic support is huge, so it is difficult to carry out in short order for some less developed areas. The more important is that the metabolic process of fluorous is long (months to several decades), so most patients' clinical symptoms are still obvious in a long time except for very few ones, and these symptoms affect their quality of life seriously. According to the theory that kidney controls bone and marrow and presides urination and defecation and patients' clinical symptoms, traditional Chinese medicine has achieved obvious effect on skeletal fluorosis aiming at regulating kidney to reinforcing bone and promoting the metabolism of fluorosis.

Skeletal fluorosis is a disease manifested as injuries in skeletons and soft tissue caused by excessive intake of fluoride for a long time and high content of fluorine in environment [6]. Main symptoms include painful and rigid joints, joint deformity, and amyotrophy. According to the theory of TCM, excessive fluoride, a kind of pathogenic factor, stays in body and causes the formation of blood stasis and phlegm. These pathogenic products block meridians and influence joints' normal movement, and pain appears when qi and blood circulation is influenced. There have been some reports on skeletal fluorosis and local kaschin-beck disease and endemic diseases treated by acupuncture in the domestic literatures. These diseases belong to Bi syndrome in TCM theory, and the treatment experience of acupuncture on Bi syndrome is rich and its clinical effect is reliable. Acupuncture is a green therapy with little side effect, and it cannot increase or reduce some materials or elements related to skeletal fluorosis directly. The use of acupuncture on skeletal fluorosis and the observation on its effect is very important for us to treat some endemic disease such as skeletal fluorosis and local kashin-beck disease.

At present, the mechanism study on acupuncture's analgesia effect on skeletal fluorosis has been very deep, and main research directions are as follows. The first is the effect of acupuncture on peripheral nerves. Experiments indicate that acupuncture or electroacupuncture on nerves conducting pain can block the conduction of pain fibers in these nerves. The second is the effect of acupuncture on nervous centralis. Acupuncture can stop new and old spinothalamic tract to conduct pain into central nervous system and then transmit relieved pain and acupuncture stimulation into different levels of central nervous system though new and old spinothalamic tract. Pain changes through the integration of neurohumor and pain modulatory system, and then the sensation and reaction caused by pain is stopped. This is the process of analgesia effect [7]. The last acupuncture can increase neurotransmitter with analgesia effect (such as ACH, 5-HT, and opioid substances in brain) or strengthen their effect and reduce neurotransmitter with antianalgesia effect (such as NA, DA) [8].

Acupuncture's effect on improving range of motion of joints is related to its analgesia effect. Studies on joint injury of skeletal fluorosis indicate that the joint dysfunction of skeletal fluorosis is caused by fibrous ankylosis actually, and this change is reversible. When pain relieves or disappears obviously, the joint dysfunction relieves or disappears too. This is the result that the tension of muscle, tendon, ligament, and so forth around joints gets lower after pain relieving [4].

Besides relieving pain of joints, the treatment group can also increase urinary fluoride, while the effect in control group is not significant statistically. This indicates that acupunctures' effect on skeletal fluorosis may be related to fluorine's excretion through urine. The level of serum calcium and phosphorus in treatment group also reduced obviously, while only the level of serum calcium in control group reduced. Calcium and phosphorus, normal elements in bone, form calcium-phosphorus metabolic pathway with effects of PTH, CT, vitamin D, and so forth, and calcium-phosphorus metabolism is closed related to osteolysis and osteogenesis [1]. Based on RCT designing methods, this clinical study indicated that acupuncture can influence serum calcium, serum phosphorus and urinary fluoride, but its mechanism is unclear. So, we may study acupuncture's effect on skeletal fluorosis through its regulating effect on calcium-phosphorus metabolism and bone metabolism in the next step.

Followup was not applied in this study due to the time limitation, but followups in 6 months, 1 year, and 3 years are suggested strongly because of the slow developing course and recurrent episodes of this disease.

Considering of the therapeutic effects and side effect, we suggest acupuncture as the first choise for the endemic skeletal fluorosis, especially for relieving the pain caused by it.

## 4. Conclusion

Both acupuncture and western medicine can relieve the pain of skeletal fluorosis patients, and the effect of acupuncture is better than that of western medicine.

The urinary fluoride of skeletal fluorosis patients increases after acupuncture treatment, while that does not change after taking western medicine.

Both acupuncture and western medicine can decrease the serum calcium of skeletal fluorosis patients, and acupuncture can decrease the serum phosphorus also.

## Figures and Tables

**Table 1a:** (a)

Group	Cases	Water fluoride ($\overset{-}{X}\pm \text{SD})$	*Z*	*P*
Treatment group	68	4.48 ± 1.44	−1.002	−0.316
Control group	31	4.88 ± 1.73		

**Table 1b:** (b)

Group	Cases	Time of residence ($\overset{-}{X}\pm \text{SD}$)	*Z*	*P*
Treatment group	68	44.21 ± 16.72	−0.151	0.88
Control group	31	44.65 ± 17.44		

**Table 1c:** (c)

Group	Cases	Labor intensity ($\overset{-}{X}\pm \text{SD}$)	*Z*	*P*
Treatment group	68	1.75 ± 0.72	−1.22	0.219
Control group	31	1.58 ± 0.77		
